# A retrospective study on LDL-C goal attainment in readmitted hypertriglyceridemia patients: risk factor analysis

**DOI:** 10.3389/fendo.2025.1553173

**Published:** 2025-08-13

**Authors:** Yuan Ji, Arsheen Chadha, Abhash Krity, Shenglan Huang

**Affiliations:** ^1^ Changzhou Medical Centre, Nanjing Medical University, Changzhou, Jiangsu, China; ^2^ Department of Cardiology, Affiliated Changzhou No.2 People’s Hospital of Nanjing Medical University, Changzhou, Jiangsu, China; ^3^ School of International Education, Nanjing Medical University, Nanjing, Jiangsu, China

**Keywords:** low-density lipoprotein cholesterol goal, trajectory, lipid-lowering therapy, dyslipidemia, retrospective study

## Abstract

**Objective:**

Despite positive impacts of lipid-lowering therapies (LLTs), the low-density lipoprotein cholesterol (LDL-C) target attainment remains suboptimal. This study aimed to investigate LDL-C goal achievement per the 2023 China guideline for lipid management among rehospitalized hypertriglyceridemia patients, who had higher chances to access the knowledge associated with lipid management and treatment, and evaluate the risk factors for LDL-C.

**Methods:**

This retrospective study was performed among rehospitalized hypertriglyceridemia patients between July 2020 and May 2023. The department-specific latent class trajectory modeling was implemented to assess the longitudinal lipid profiles. The risk factors of goal attainment were evaluated using multivariate Cox regression analysis.

**Results:**

Among 8905 readmitted patients, 5045 (56.7%) had two admissions. Only 27.1% consistently achieved LDL-C targets, while 25% never did. Nearly half were eligible for LLTs, but only 25% received them. Continuous LLT use was associated with higher goal attainment (HR: 1.23 [95% CI: 1.12–1.36]). Most readmissions (92.15%) had increasing LDL-C trajectories and less odds of achieving the LDL goals at the last hospitalization. At the latest hospitalization, patients with higher atherosclerotic cardiovascular disease (ASCVD) risk had higher chances of achieving their LDL-C targets (hazard ratio 2.00 [95% CI, 1.70-2.36]).

**Conclusions:**

LDL-C control remains poor in this population. Continuous LLT use and ASCVD risk stratification are important factors for goal attainment, highlighting the need for better long-term management and closer monitoring of low-risk patients.

## 1 Introduction

Cardiovascular disease (CVD) is the leading cause of death in China, affecting over 330 million individuals (1). In 2019, CVD caused 46.7% and 44.3% of deaths in rural and urban areas in China, respectively (1). Atherosclerotic cardiovascular disease (ASCVD) is the major subset of CVD caused by deposition of plaques in the arteries, involving conditions including coronary artery disease, cerebrovascular disease, and peripheral artery disease (2). Notably, the formation of these plaques is directly related to excess lipids in the circulation, such as low-density lipoprotein cholesterol (LDL-C) and triglycerides; hence, hypercholesterolemia and hypertriglyceridemia are risk factors for ASCVD, while reaching the LDL-C target is the primary goal in ASCVD risk intervention. The American Heart Association incorporated blood lipids as one of the elements in ‘Life’s Essential 8’, an important guideline for maintaining cardiovascular health, to form a novel composite cardiovascular health score (3). The latest China guideline for lipid management has also redefined the new ASCVD risk stratification and updated more stringent LDL-C control goals accordingly (4).

Unfortunately, achieving the appropriate levels of LDL-C remains a challenge in clinical practice. The DA VINCI study showed that 60.4% of enrolments did not achieve guideline-recommended risk-based LDL-C goals (5). Moreover, in patients with Type 1 diabetes mellitus (DM), only 6.16% reached the recommended LDL-C goal, while 11.81% reached the goal in Type 2 DM (6), and the LDL-C goal attainment rate of DM increased to 30.2% in six months (7). A multicenter observational study indicated that patients who suffered from their first major acute cardiovascular event (MACE) while on statin therapy were undertreated, and only 10% of the patients at very high cardiovascular risk reached their lipid targets (8). Another recent report also showed that among patients undergoing coronary artery bypass grafting, only 14.9% could achieve the guideline-recommended goals after one year (9).

The gap between guidelines and clinical practice calls for concern. In this retrospective study, we aim to investigate the LDL-C goal attainment among rehospitalized hypertriglyceridemia patients and to reveal the risk factors associated with successful or unsuccessful lipid management. We choose these patients because 1) we aim to investigate the trajectory of lipid profiles of these patients, which required at least two valid LDL-C measurements, and 2) these patients may have higher chances to access the knowledge associated with lipid management, as well as treatment; therefore, this cohort may better highlight the gap between guideline and practice. This study will describe the clinical characteristics of these patients, elaborate on the prescription rate of lipid-lowering therapies (LLTs), and assess the LDL-C goal attainment according to the 2023 China guidelines for lipid management. In addition, we contoured the trajectories of LDL-C and evaluated the risk factors of LDL-C goal attainment before the last admission.

## 2 Methods

### 2.1 Study design and population

This single-centered, retrospective study collected in-hospital information through the electronic health record system from July 2020 to May 2023. The inclusion criteria are 1) patients with complete medical records, including lipid profile (at least one valid LDL-C measurement during each admission), age, comorbidities (e.g., diabetes and hypertension), and previous history of ASCVD; 2) triglycerides (TG) were more than 1.7 mmol/L (150 mg/dl); 3) admitted twice or more than twice. The exclusion criteria are: 1) patients admitted to pediatrics, obstetrics, and neonatology departments; 2) TG below 1.7 mmol/L; and 3) admitted only once.

### 2.2 Data collection and definition

According to the latest guideline (4), the patients were classified into hierarchical ASCVD risk groups: low-risk, medium and high-risk, very high risk, and ultra-high risk. The target values for LDL-C vary based on these ASCVD risk classes (low risk: less than 130 mg/dl, medium and high risk: less than 100 mg/dl, very high risk: less than 70 mg/dl and more than 50% reduction from baseline, ultra-high risk: less than 55 mg/dl and more than 50% reduction from baseline) (4). For each patient, risk evaluation was performed independently using the data collected at every admission. The risk group at the first admission, and the lowest and highest risk stratifications for each patient were recorded for analysis.

Considering that physicians in some departments are more likely to treat patients with ASCVD, more accessible to lipid-lowering guidelines, and may have a better acquirement and implementation of the latest lipid management guidelines, we categorized all departments into either ASCVD-related departments, which had a higher incidence of ASCVD (including departments of cardiology, cardiac surgery, neurology, endocrinology, nephrology, vascular surgery, geriatrics, rheumatology, and general practice), or non-ASCVD related departments (departments other than the above).

### 2.3 Statistical analysis

All analyses were performed using the R software (version 4.1.1; R Foundation for Statistical Computing, Vienna, Austria). Diagrams were plotted using R package ggplot2(3.4.1), RcppCGAL, WeightedTreemaps, Waffle, corrplot, and Forestplot (V3.1.3). Continuous variables were expressed as mean ± SD or median (interquartile ranges), and Student’s t-tests or chi-square tests were used to compare two groups. Categorical variables were expressed as frequency and proportion and were compared using the chi-square test. The Holm-Sidak *post hoc* test was used for repeated comparisons between multiple groups.

To illustrate the prevalence of LLTs prescription against different lipid profile backgrounds, a correlation plot was used to calculate the proportion among readmissions. Considering the non-independence of the lipid profile from repeated hospitalizations, we implemented mixed-effects models to explore the trajectories in continuous variables at different times. Finally, department-specific latent class trajectory modeling (LCTM) for LDL-C was used. The chosen number of models was based on the lowest Bayesian information criterion while maintaining the posterior probabilities by class (>0.7) and class size (more than 2% of the population). We calculated the posterior probabilities of patients for each trajectory and assigned participants to the trajectory with the highest probability. The Cox proportional hazards model was used to predict LDL-C goal-attainment factors. A forest plot of the likelihood of reaching the LDL-C target among patients during their last hospitalization was generated based on adjusted hazard ratios derived from the constructed multivariate model. A two-sided P value less than 0.05 was considered statistically significant.

## 3 Results

### 3.1 Baseline characteristics

**Figure 1:**
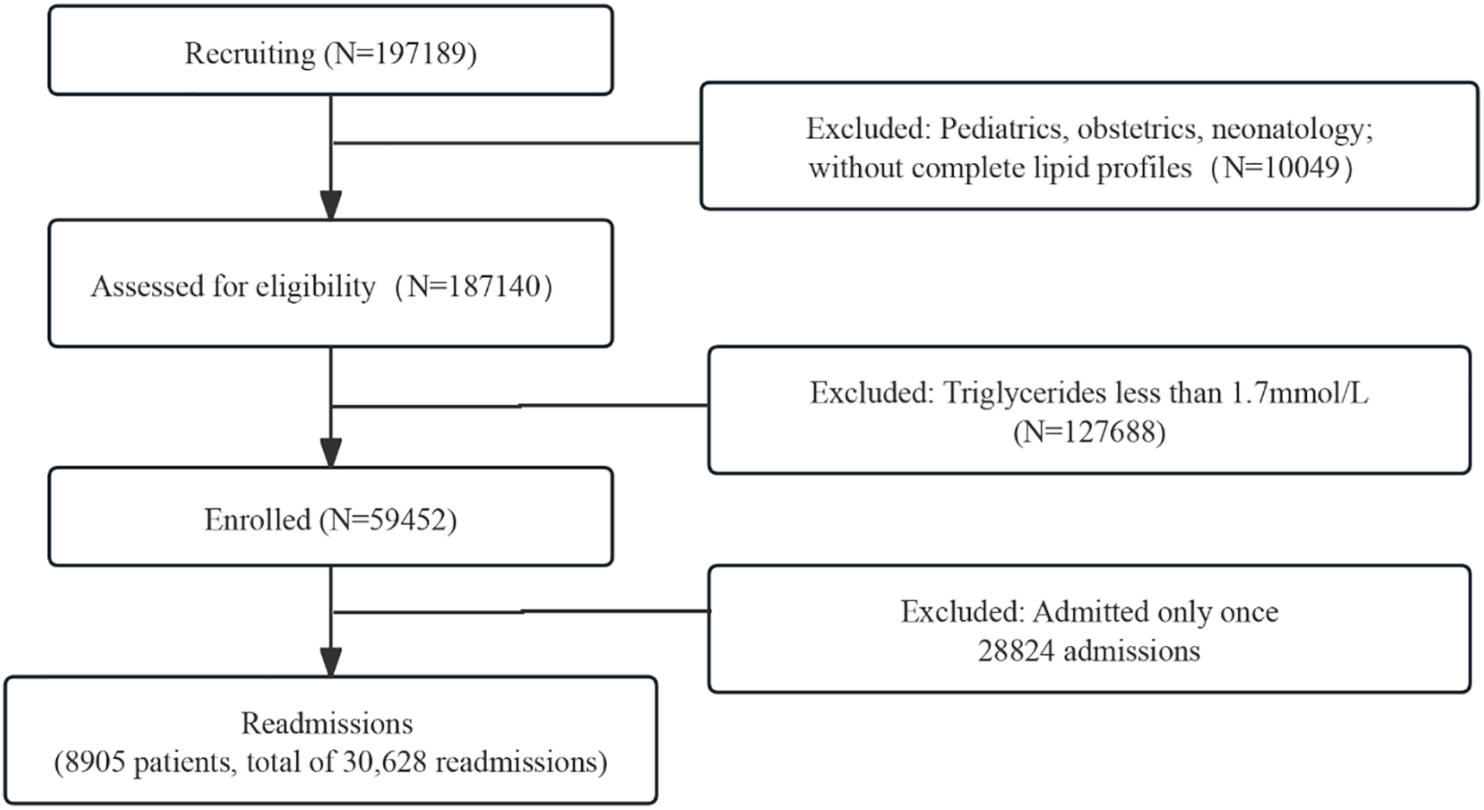
The study flowchart. A total of 59,452 hypertriglyceridemia patients were enrolled, and 8,905 readmitted patients were finally included based on complete lipid profile data and readmission criteria.

The overall screening procedure is shown in **Figure 1**. In total, the analysis included 59,452 hypertriglyceridemia patients. The mean age of all patients was 57.53 ± 14.37 years and males accounted for 52.17%. The baseline characteristics of all patients are shown in **Table 1**.

**Table 1:** Baseline characteristics.

Baseline characteristics	Total (N=8905)	LLTs never prescribed (N=6074)	LLTs once prescribed (N=2281)	LLTs always prescribed (N=550)	p
Male-no. (%)	4560 (51.2)	3018 (49.7)	1200 (52.6)	342 (62.2)	<0.001
Duration (d)- (IQR)	306 (92,547)	274 (91,516)	426 (212,639)	243 (61,426)	<0.001
Diabetes-no. (%)	1074 (12.1)	475 (7.8)	430 (18.9)	169 (30.7)	<0.001
Hypertension-no. (%)	4964 (55.7)	3008 (49.5)	1487 (65.2)	469 (85.3)	<0.001
Chronic renal dysfunction-no. (%)	659 (7.4)	296 (4.9)	264 (11.6)	99 (18.0)	<0.001
Coronary artery disease-no. (%)	108 (1.2)	0 (0)	52 (2.3)	56 (10.2)	<0.001
Stroke-no. (%)	406 (4.6)	83 (1.4)	208 (9.1)	115 (20.9)	<0.001
Peripheral artery disease-no. (%)	32 (0.4)	2 (0)	11 (0.5)	19 (3.5)	<0.001
LDL-C meet the target-no. (%)					
Never	2214 (24.9)	1294 (21.3)	614 (26.9)	306 (55.6)	<0.001
Once not	4281 (48.1)	2758 (45.4)	1347 (59.1)	176 (32.0)	
Always	2410 (27.1)	2022 (33.3)	320 (14.0)	68 (12.4)	
Risk stratification shift	4543 (51.0)	2744 (45.2)	1579 (69.2)	220 (40.0)	<0.001
Lowest risk stratification-no. (%)					
Low risk	5842 (65.6)	4416 (72.7)	1327 (58.2)	99 (18.0)	<0.001
Moderate risk	1083 (12.2)	738 (12.2)	296 (13.0)	49 (8.91)	
High risk	1674 (18.8)	879 (14.5)	579 (25.4)	216 (39.3)	
Very high risk	80 (0.9)	21 (0.35)	23 (1.01)	36 (6.55)	
Ultra-high risk	226 (2.5)	20 (0.33)	56 (2.46)	150 (27.3)	
Highest risk stratification-no. (%)					
Low risk	2388 (26.8)	2173 (35.8)	197 (8.64)	18 (3.27)	<0.001
Moderate risk	1354 (15.2)	1135 (18.7)	198 (8.68)	21 (3.82)	
High risk	3880 (43.6)	2555 (42.1)	1119 (49.1)	206 (37.5)	
Very high risk	303 (3.4)	85 (1.40)	180 (7.89)	38 (6.91)	
Ultra-high risk	980 (11.0)	126 (2.07)	587 (25.7)	267 (48.5)	
LDL-C (mg/dl)- (IQR)					
minimum	91.3 (74.2, 110.6)	94.0 (77.0, 112.1)	86.6 (70.4, 106.3)	82.4 (67.7, 104.4)	<0.001
maximum	129.2 (109.0, 150.4)	127.6 (108.3, 147.3)	136.1 (114.8, 161.6)	118.9 (97.1, 145.3)	<0.001
Total cholesterol (mg/dl)- (IQR)					
minimum	164.1 (141.7, 187.6)	167.5 (145.5, 189.5)	157.5 (135.9, 182.6)	149.8 (130.1, 177.5)	<0.001
maximum	211.9 (186.4, 242.4)	210.4 (186.1, 238.9)	220.4 (192.2, 254.8)	194.7 (166.9, 232.4)	<0.001
HDL-C (mg/dl)- (IQR)					
minimum	42.6 (34.6, 51.6)	43.8 (35.9, 52.3)	40.4 (32.8, 49.6)	38.4 (31.6,48.7)	<0.001
maximum	60.2 (50.8,70.1)	59.5 (50.5, 68.7)	63.5 (53.5,75.4)	55.4 (45.3, 67.8)	<0.001
Triglycerides (mg/dl)- (IQR)					
minimum	46.3 (40.5, 54.8)	46.7 (40.5, 54.8)	46.7 (40.5, 56.0)	40.9 (35.9, 47.5)	<0.001
maximum	170.8 (158.4, 199.1)	171.7 (158.4, 199.1)	169.0 (157.5, 195.6)	179.7 (162.0, 212.4)	<0.001
Lp (a) (mg/dl)- (IQR)					
minimum	8.0 (0.0, 19.0)	8.0 (0.0, 18.0)	10.0 (1.0, 20.0)	8.0 (0.0, 22.0)	<0.001
maximum	13.0 (0.0, 28.0)	12.0 (0.0, 26.0)	17.0 (5.0, 37.0)	10.0 (0.0, 26.8)	<0.001

LLTs, lipid-lowering therapies; LDL-C, low-density lipoprotein cholesterol; HDL-C, high-density lipoprotein cholesterol; Lp (a), lipoprotein (a); IQR, interquartile range.

### 3.2 Clinical characteristics of hypertriglyceridemia among readmissions

**Figure 2:**
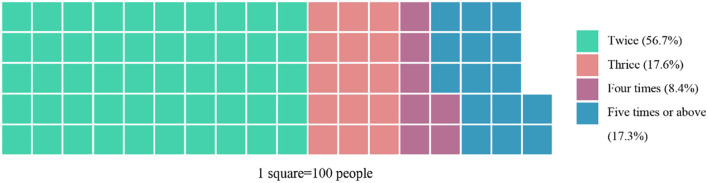
Distribution of readmission frequency. More than half of the patients (56.7%) were admitted twice, with a notable portion (25.7%) admitted more than three times.

Among 8905 readmitted patients diagnosed with hypertriglyceridemia, 5045 (56.7%) of them were discharged twice, 1563 (17.6%) were discharged three times, and 1577 (17.7%) were discharged more than five times (**Figure 2**). Among these patients, 1074 (12.1%) and 4964 (55.7%) were diagnosed with DM and hypertension, respectively. A total of 5492 (61.7%) patients were discharged from the same department, whereas 960 (17.5%) were from ASCVD-related departments (i.e., had at least one hospitalization at any of the ASCVD-related departments). The top reason for readmission was carcinoma-related, accounting for 38%, and recurrent ASCVD events requiring emergency procedures accounted for 2.4%.

### 3.3 Risk stratification re-evaluation, lipid profile, LLTs, and LDL-C goal attainment among readmissions

**Figure 3:**
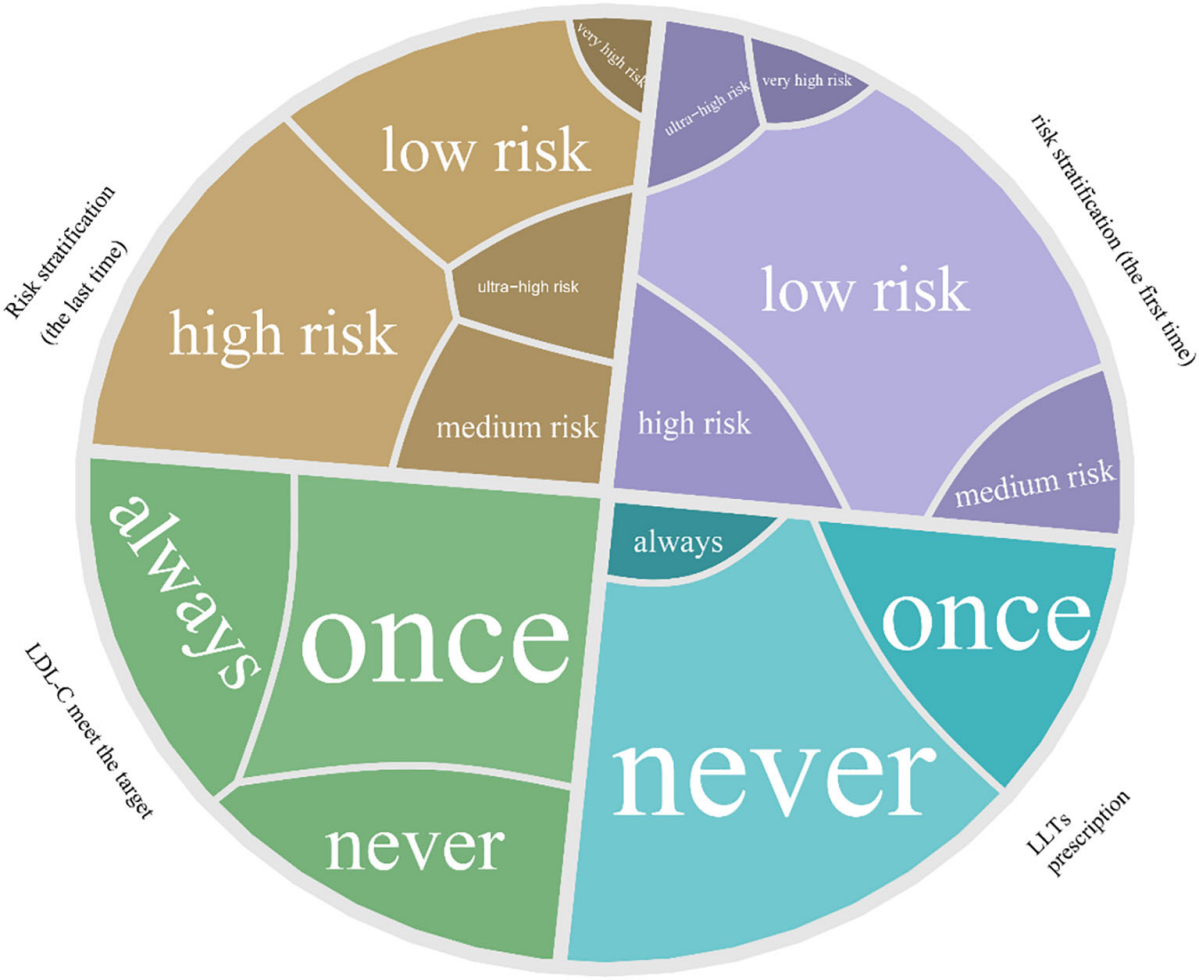
Voronoi treemap showing dynamic changes in ASCVD risk stratification at the first and last time of admission, LLT prescription, and LDL-C goal attainment across readmissions. Each cell represents a patient subgroup defined by their stratification change and treatment pattern. Area size corresponds to the number of patients in each subgroup. LLTs, lipid-lowering therapies; LDL-C, low-density lipoprotein cholesterol.

During the readmissions, 51% of the patients were upgraded to a higher category compared with the first admission. The shift in ASCVD risk stratification, the prescription of LLTs, and the LDL-C goal attainment during readmissions are shown in a Voronoi treemap (**Figure 3**). The specific percentages are presented in **Table 1**. Notably, the number of patients in the ultra-high-risk group was more than tripled, from 226(2.5%) to 980(11.0%) after re-evaluation. Despite their ASCVD risk, 68.2% of the readmitted patients were never prescribed LLTs; 24.9% of the readmitted patients never met their LDL-C target, while only 27.1% consistently achieved the target according to the Chinese guidelines. Even for the patients who were prescribed LLTs continuously, only 12.4% of them kept reaching the optimal LDL-C target level (**Table 1**). It is also important to note that, compared with other groups, the group prescribed LLTs had significantly lower minimum and maximum LDL-C and TC values, as well as lower minimum TG values (**Table 1**).

**Figure 4:**
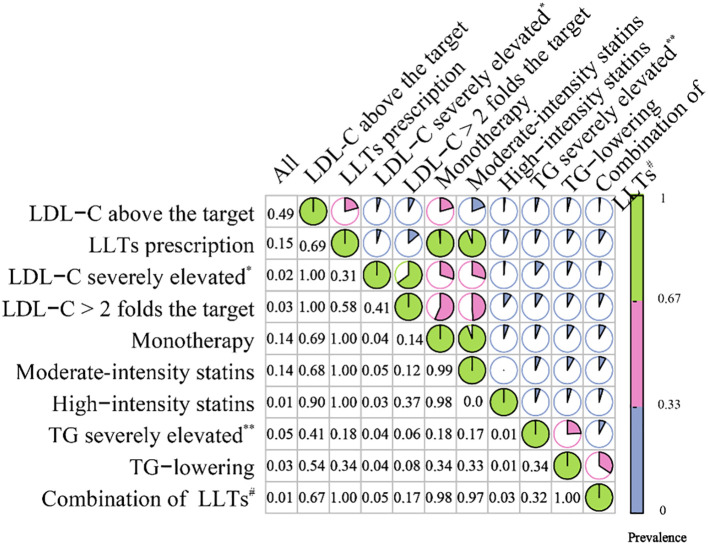
Correlation matrix plot for the cross-tabulation among LLTs and lipid profile. Rows represent conditions such as elevated LDL-C or TG; columns show their co-occurrence rates. The size and intensity of the colored circles indicate the strength of co-occurrence. LLTs, lipid-lowering therapies; LDL-C, low-density lipoprotein cholesterol *LDL-C>4.9 mmol/L (189 mg/dl); **TG more than 5.6 mmol/L (496 mg/dl); #combination of TG and LDL-C lowering therapies. Correlation matrix plot for the cross-tabulation among LLTs and lipid profile. The figure reports the prevalence of each condition (rows) among the overall readmissions and among each other condition (columns).

We then performed a cross-tabulation analysis between the LLTs and lipid profiles of the patients (**Figure 4**, **Supplementary Data 1**). A total of 48% of the readmitted patients met the indications for receiving LLTs; however, only 25% of them were finally prescribed. Even for those with severely elevated LDL-C levels (LDL-C more than 4.9 mmol/L or 189 mg/dl), only 35% were prescribed. This proportion increased to 65% among readmissions when LDL-C was more than two times the guideline-recommended level. Most LLTs relied on monotherapy (99%), particularly moderate-intensity statins (94%). Only 9% of the patients with very high LDL-C levels (more than two times over the guideline-recommended level) were prescribed high-intensity statins. Meanwhile, 15% of the readmissions with severely elevated LDL-C levels had concomitant severely elevated TG levels (TG more than 5.6 mmol/L or 496 mg/dl); however, even among these mixed dyslipidemia patients, only one-fifth were prescribed combination therapy consisting of both LDL-C and TG-lowering medications.

### 3.4 Trajectories among readmissions

**Figure 5:**
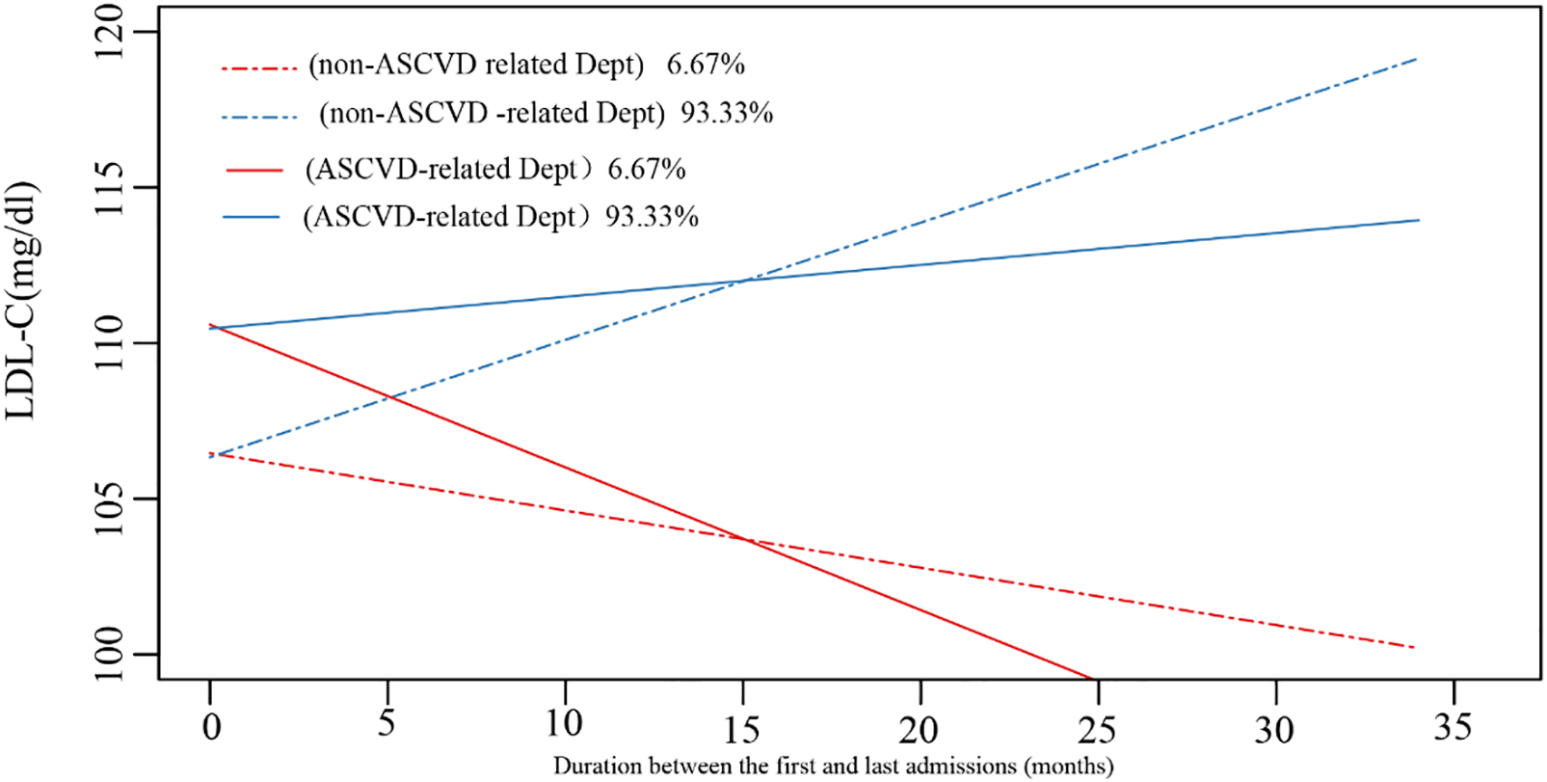
The trajectories of LDL-C profile among readmissions. ASCVD, atherosclerotic cardiovascular disease; Dept, department.

We implemented the LCTM analysis to contour the lipid trajectories and determine the LDL-C changes among readmissions. The trajectories were divided into two classes, considering the departments, LLT frequencies, and risk stratification (**Figure 5**). Patients were stratified into ASCVD-related departments (solid lines) and non-ASCVD-related departments (dotted lines). Most patients (n=8311, 93.33%) were classified as Class 2 (the blue line) with an increasing LDL-C trajectory, while the others were classified as Class 1 (the red line) with a decreasing trajectory.

Only 51.8% of all patients achieved the target level during their last admission, and the percentage of patients reaching the target level was 82.5% and 49.6% in Class 1 and Class 2, respectively, during their latest admission. Class 2 trajectory was mainly seen in low, moderate, and high-risk groups, while class 1 was only seen in very high and ultra-high-risk groups. Compared to Class 2, patients in Class 1 showed lower levels of minimum and maximum LDL-C, HDL-C, and TC. Neither the minimum nor the maximum level of TG demonstrated the difference between the two groups. The minimum Lp(a) in the Class 1 group was higher than in the Class 2 group, while the maximum level showed no difference (**Table 2**).

**Table 2:** Clinical characteristics between different trajectories among readmissions in all departments.

Clinical characteristics	Total (N=8905)	Class 1 (decreasing trajectory) N=594	Class 2 (increasing trajectory) N=8311	p
LDL-C achieved the target level during the last hospitalization-[no. (%)]	4613 (51.8)	490 (82.5)	4123 (49.6)	<0.001
LLTs prescription during the last hospitalization [no. (%)]	1708 (19.2)	381 (64.1)	1327 (16)	<0.001
Number of times LLTs were prescribed before last hospitalization (IQR)	0 (0,0)	1.0 (0,1)	0 (0,0)	<0.001
Total number of times LLTs were prescribed (IQR)	0 (0,1)	1 (1,2)	0 (0,1)	<0.001
The highest risk stratification [no. (%)]				
Low risk	2388 (26.8)	0 (0)	2388 (28.7)	
Moderate risk	1354 (15.2)	0 (0)	1354 (16.3)	
High risk	3880 (43.6)	0 (0)	3880 (46.7)	<0.001
Very high risk	303 (3.4)	59 (9.9)	244 (2.9)	
Ultra-high risk	980 (11.0)	535 (90.1)	445 (5.4)	
Max LDL-C [mg/dl (IQR)]	129.2 (109.0,150.4)	123.4 (97.8,144.2)	129.5 (109.8,150.8)	< 0.001
Min LDL-C [mg/dl (IQR)]	91.3 (74.2, 110.6)	78.9 (67.1,99.2)	92.4 (75.0, 111.4)	< 0.001
Max TG [mg/dl (IQR)])	260.2 (209.7,354.0)	259.3 (207.1,338.1)	260.2 (209.7,355.8)	0.202
Min TG [mg/dl (IQR)]	170.8 (158.4,199.1)	171.7 (158.4,195.1)	170.8 (158.4,199.1)	0.533
Max HDL-C [mg/dl (IQR)]	46.3 (40.5, 54.8)	42.5 (36.9, 50.0)	46.7 (40.5, 55.2)	< 0.001
Min HDL-C [mg/dl (IQR)]	34.7 (29.3, 40.5)	32.0 (27.4, 37.4)	35.1 (29.3, 40.9)	< 0.001
Max TC [mg/dl (IQR)]	211.9 (186.4,242.4)	196.1 (168.3,229.7)	213.1 (188.0,243.2)	< 0.001
Min TC [mg/dl (IQR)]	164.1 (141.7,187.6)	146.3 (127.4,167.7)	165.6 (143.2,188.8)	< 0.001
Max Lp (a) [mg/dl (IQR)]	20.0 (10.0, 40.0)	20.0 (10.0, 40.0)	20.0 (10.0, 40.0)	0.051
Min Lp (a) [mg/dl (IQR)]	10.0 (10.0, 20.0)	20.0 (10.0, 30.0)	10.0 (10.0, 20.0)	< 0.001

HDL-C, high-density lipoprotein cholesterol; LLTs, lipid-lowering therapies; LDL-C, low-density lipoprotein cholesterol; TG, Triglyceride; TC, total cholesterol; Lp (a), Lipoprotein (a); Max, maximum; Min, minimum; IQR, interquartile range.

### 3.5 Risk factors for LDL-C goal attainment

**Figure 6:**
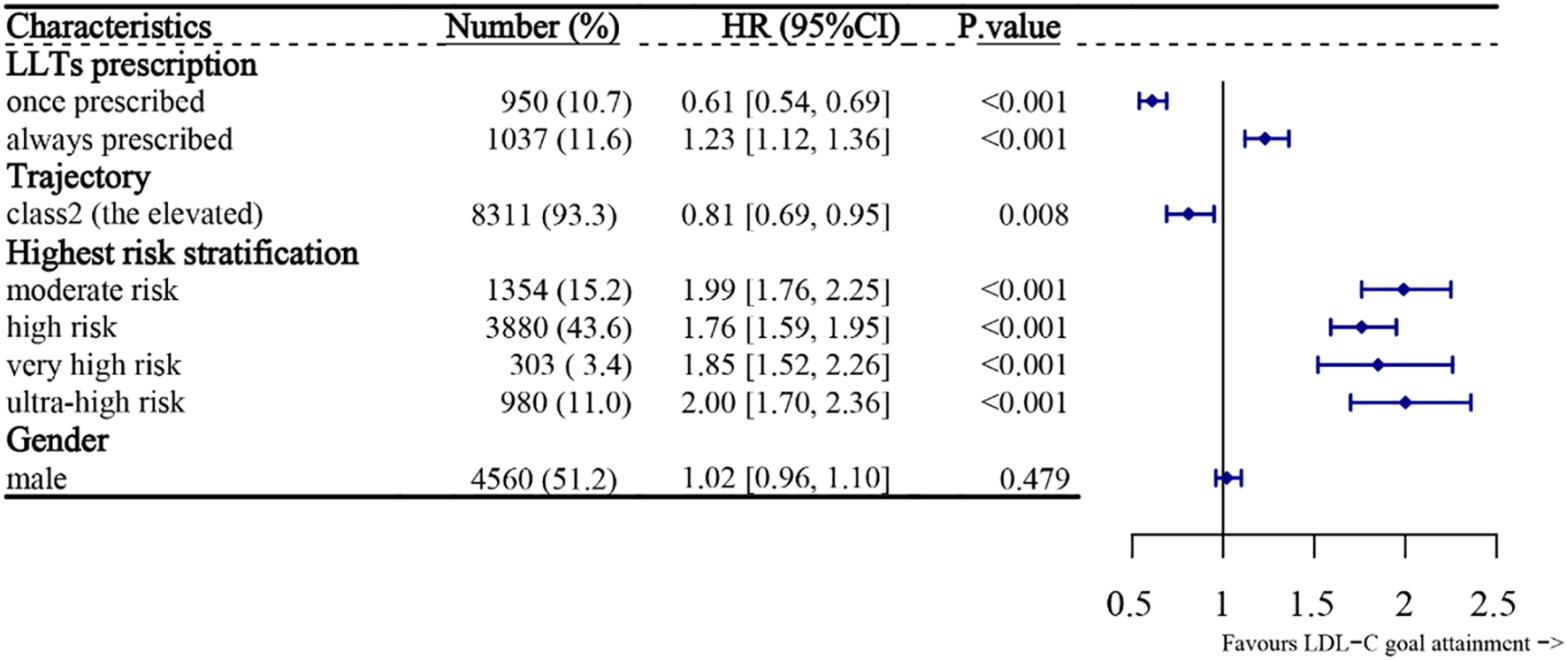
Forest plot for the predictors and LDL-C goal attainment during the last hospitalization. Factors include LLT prescription continuity, LDL-C trajectory, and highest ASCVD risk level. HRs and 95% confidence intervals are shown. Values >1 indicate higher likelihood of goal attainment. LLTs, lipid-lowering therapies; LDL-C, low-density lipoprotein cholesterol; HR, hazard ratio; CI, confidence interval.

Factors influencing LDL-C treatment control were analyzed using the multivariate COX regression. Due to the unfulfilled COX proportional hazard assumptions, age and the ASCVD-related departments’ hospitalization rate were treated as stratification factors. The model included LLTs proportions (before the last admission), gender, LDL-C trajectories, and the highest risk stratification. **Figure 6** shows that the patients who were always prescribed LLTs had 23% [HR: 1.23 (95%, 1.12-1.36)] higher chances of attaining the LDL-C goals in the last readmission compared to the never prescribed ones, and those with increasing trajectories had 19% [HR: 0.81 (95%, 0.69-0.95)] lower chance of LDL-C goal-attainment, compared to the group with decreasing trajectories. Compared with the low-risk group, other groups achieved better goal attainment. Notably, the ultra-high-risk group had a goal-attainment rate twice that in the low-risk group [HR: 2.00 (1.70,2.36), p<0.001].

## 4 Discussion

### 4.1 Main interpretation

This study has revealed poor LDL-C target attainment among the readmitted hypertriglyceridemia patients. Nearly half of the readmissions with hypertriglyceridemia were indicated for LLTs (excluding those with obvious contraindications, such as liver or renal dysfunction), but only 25% of them were finally prescribed, whereas about one-fourth of the total readmissions never met their LDL-C target. Even for the patients prescribed LLTs continuously, only 12.4% kept reaching the optimal target. Still, patients with continuous LLT before the last visit had significantly better target attainment than those without prescribed LLT. Further analysis revealed that over 90% of the patients showed an increasing LDL-C trajectory since the first admission. To better understand this phenomenon, we further analyzed possible causes including insufficient LLT prescription rates, underuse of high-intensity or combination therapies, and lack of treatment adjustment based on risk reassessment.

This study suggests a significant awareness gap between clinical practice and guidelines, as LLT prescription is far less than expected, and LDL-C goal attainment is suboptimal. Previous studies have shown that among acute coronary syndrome patients, LDL-C goal attainment at discharge was less than 20% and increased to 38%-64% in 12 months (10), which was consistent with our results: after a median follow-up of 306 days, 51.8% of patients achieved the LDL-C target. Other previous studies also affirmed this (11–13), which is even worse in middle-income regions (14, 15). These results suggest that lack of intensification of LLTs and inadequate combined LLT medication may be important factors contributing to the low rates of LDL-C goal attainment.

In order to reduce the overall risk of ASCVD, guidelines have lowered the LDL-C management goal thresholds, aiming to cover more patients using LLTs prescription. The 2022 American College of Cardiology (ACC) expert consensus set the LDL-C standard at less than 55 mg/dl (1.4mmol/L) with at least 50% reduction from baseline for adults in very high-risk groups (16). It was also recommended that this goal be achieved by a combination with non-statin therapy, such as ezetimibe or a proprotein convertase subtilisin/kexin type 9 inhibitor (PCSK9i). The 2019 European Society of Cardiology (ESC) and European Atherosclerosis Society (EAS) guidelines called for similar recommendations for LDL-C goals but lowered the threshold to 40 mg/dl (1.0 mmol/L) for ASCVD patients with a second event within two years while on maximally tolerated LLTs (17). However, “the lower (level of LDL-C), the better (outcome)” translates to “the lower (target according to the updated guideline recommendation), the tougher (goal attainment).” More stringent standards lead to lower LDL-C goal attainment (18), highlighting the importance of more interventions and management.

Besides LLTs prescription rate, LLTs strategy is also critical to LDL-C goal attainment. Chinese guidelines recommend moderate-intensity statin therapy as an initial prescription (4). As verified in our research, moderate-intensity statins were the most frequently prescribed LLTs covering all the ASCVD risk stratification groups. Meanwhile, in our study, high-intensity statins were only administered for about 12-13% of very high and ultra-high-risk patients, agreeing with a previous report that only 11% of patients were prescribed high-intensity statins for primary prevention (19). Another study reported that only 15% of the high or very high-risk groups received high-intensity statins at the time of their first MACE (8). However, it should be emphasized that most patients in the high ASCVD risk group require combined high-intensity LLTs, which may result in greater reduction in LDL-C (5); also, high-intensity statins are assumed to lower the LDL-C by more than 50%. We also found that statin monotherapy was the most prescribed, consistent with other literature (7, 13). These results suggest that lack of intensification of LLTs and inadequate combined LLT medication may be important factors contributing to the low rates of LDL-C goal attainment. In addition, the under-prescription of LLTs may be attributed to the lack of guideline awareness in non-ASCVD departments and limited access to non-statin therapies. It is also worth noting that non-statin therapies may serve as an effective alternative for statin intolerant patients, who may discontinue statin-based LLT due to its side effects (20). Similar opportunities for improvement have also been highlighted in coronary artery disease populations, emphasizing the need for intensification of lipid-lowering strategies and improved multidisciplinary management (21).

Another prominent factor contributing to the undermanagement of LDL-C levels may be therapeutic delay and nonadherence to medication. The present research concluded that continuous prescription of LLTs might contribute to higher goal achievement, while no significant protective effect was observed for those with LLTs prescribed only once. Nonadherence to medication may result from a general lack of public awareness, i.e., of both physicians and patients, of the management and therapy suggested by the guidelines. Improving physicians’ compliance with guidelines (22) and patients’ adherence may improve low LDL-C goal attainment, suggesting an urgent need for a comprehensive, cross-disciplinary guide for physicians and a detailed instruction oriented toward patients. Also, the fact that patients at low risk for ASCVD had lower LDL-C goal-attainment rates than other groups suggested that this broader population may be overlooked. Physicians may need to improve their awareness and embrace cross-disciplinary cooperation for better LDL-C management.

The present study also emphasizes the importance of dynamic evaluation and adjustment among readmissions, including ASCVD risk stratification and assessment of treatment efficacy. More than half of readmissions shifted to higher risk stratification, whereas less than 15% kept reaching the LDL-C goal even under continuous LLTs. Failure to adjust the treatment regimens (including combination with non-statin medications or upgraded statin intensity) promptly based on reassessed risk stratification may be a nonnegligible factor for the undermanagement of dyslipidemia. Previous studies showed gender disparities in LDL-C goal attainment: women were less likely to attain LDL-C goals than men (13, 23). However, our present study showed that the prescription continuity of LLTs and the decreased trajectory were the main factors promoting goal attainment, while gender showed no significance. The effect of gender on LDL-C goal attainment may be associated with differences in the societal roles and dietary habits of male and female patients in different areas of China, which is worth further investigation.

In the present study, readmissions exemplified two trends: the “stably increasing” and “moderately decreasing” trajectories in non-ASCVD-related departments and the “moderately increasing” and “stably decreasing” trajectories in ASCVD-related departments. The increasing trajectory predominated among the longitudinal cohort with a median follow-up of 306 days and is associated with poorer LDL-C goal attainment compared with the group with the decreasing trajectory. The group with decreasing trajectory is mainly composed of patients with high ASCVD risks at their first admissions; therefore, these patients were more likely to be treated early and continuously, resulting in higher chances to reach their LDL-C goals. In addition, the clinicians in ASCVD-related departments are more likely to focus on the patients’ lipid management, emphasizing LLT prescription, close lipid monitoring, and patient education, since lipid management is critical to the management of their primary diseases (e.g., stroke and coronary heart disease). These clinicians may also be more experienced in treating patients with high risks of hypertriglyceridemia and/or tend to strictly follow the guidelines, which is consistent with a previous study (24). In contrast, the clinicians in non-ASCVD-related departments may focus more on other primary diseases that led to hospitalization, leading to reduced focus on lipid management. To eliminate this disparity, multidisciplinary team-based care and shared decision-making may be effective in promoting the awareness of both patients and clinicians across different departments (25). Previous studies also applied trajectory analysis on lipid profiles: Finnish type 2 DM cohort showed that the majority of patients (85.9%) had relatively stable LDL-C levels, around 2.3 mmol/L (89 mg/dl) (26). A community-based US cohort study showed a decreasing LDL-C trajectory in approximately 80% of the enrollments (27). The disparities between our study and previous studies may result from different enrollment strategies, trajectory approaches, and the follow-up duration.

To the best of our knowledge, this is the first retrospective research on the LDL-C goal attainment among readmitted hypertriglyceridemia patients according to the updated China guidelines for lipid management. This study utilized different target levels of lipid criteria corresponding to the ASCVD risk stratification. Considering the variations in guideline recommendations across other regions, country-specific recommendations regarding hyperlipidemia screening and LDL-C goal thresholds may be appropriate when analyzing the regional conditions (28). Few previous studies have focused on LDL-C goal achievement for readmissions. The findings in this study provide a more concise estimation of the dyslipidemia problem by analyzing longitudinal data on readmissions. Also, we utilized trajectory analysis to illustrate dynamic patterns over time and provide more comprehensive information with repeated measurements than a baseline parameter or average level. Furthermore, we employed the LCTM method to identify heterogeneity in the LDL-C profile, which the conventional approach cannot fulfill. In addition, while elevated TG levels may be correlated to ASCVD risks, previous clinical trials controlling TG levels with fibrates did not significantly reduce ASCVD outcomes (12–14). Moving forward, future studies should include multicenter, prospective cohorts and incorporate direct LDL-C measurement for improved accuracy. Furthermore, systematic educational interventions, discharge LDL-C checklists, and cross-departmental collaboration may help improve LLT implementation and LDL-C goal attainment.

### 4.2 Limitations

However, this study is not without limitations. Firstly, this study is constrained by its retrospective nature, though we analyzed over 8000 patients. Therefore, there is a potential selection bias (e.g., from including only readmitted patients with available LDL-C data) and information bias (e.g., misclassification or missing treatment records). The specific follow-up information, such as LLT adherence and persistence, which played an essential role in hyperlipidemia control (19, 29), was not available. Since we focused on LDL-C goal attainment, we did not analyze the management of other concomitant risk factors, such as blood pressure and glucose, which may also have cardiovascular impacts. We failed to collect data related to death as no recorded available data could be retrieved in this population. Furthermore, as this was a single-center study conducted in a general hospital, where neurology and internal medicine clinics are the primary departments managing hypertriglyceridemia, there may have been an overrepresentation of ischemic stroke patients compared to those with coronary artery disease, which could limit the generalizability of these findings to the broader ASCVD population. Secondly, LDL-C was estimated using the Friedewald equation since direct measurement was not available. Therefore, its accuracy may be affected in patients with triglyceride levels above 4.52 mmol/L (400 mg/dL). In our dataset, 19.3% of patients had a maximum TG value exceeding this threshold during at least one admission, which could introduce potential bias in LDL-C estimation. In future studies, we will consider incorporating direct LDL-C measurements to improve accuracy. Thirdly, many non-statin LLTs were unavailable to most patients due to the longer approval time in China at the time of investigation. It is worth mentioning that LDL-C goal attainment may be higher with PCSK9i, small/short interfering (siRNA), and other novel medications that show more effective LDL-C reduction. Future clinical data, including new LLTs, may lead to better LDL-C goal attainment.

### 4.3 Conclusion

In conclusion, this study suggested poor LDL-C target attainment among the readmitted hypertriglyceridemia patients, which may be attributed to low LLT prescription rate and limited dose and treatment strategies. Most patients showed an increasing LDL-C trajectory during readmission, which was associated with poor LDL-C goal attainment compared with those showing a decreasing LDL-C trajectory. Efforts should be made to bridge the gap between the guidelines and clinical practice for better LDL-C goal attainment and ASCVD risk control.

## Data Availability

The original contributions presented in the study are included in the article/**Supplementary Material**. Further inquiries can be directed to the corresponding author.
